# Patient-driven surveys of mucopolysaccharidoses revealed patient-reported outcomes in the Japan MPS patient and family group

**DOI:** 10.1186/s13023-026-04479-z

**Published:** 2026-07-03

**Authors:** Toko Shibuya, Takeyuki Akiyama, Tetsuji Fujiyama, Takanobu Otomo

**Affiliations:** 1 Department of Molecular and Genetic Medicine, Kawasaki Medical School, Kurashiki, 701-0192 Japan; 2 Department of Pediatrics, University of Osaka, Suita, 565-0871 Japan; 3 Japan MPS Patient and Family Group (LysoBridge Japan), Tokyo, Japan

**Keywords:** Mucopolysaccharidosis, Patient and family group, Patient-driven survey, Patient-reported outcome

## Abstract

**Background:**

Japan Mucopolysaccharidoses (MPS) Patient and Family Group (J-MPS) was founded in 1986. It is the largest patient group for all types of MPS in Japan and now includes some of the patients with lysosomal storage diseases. Patients receive medical care but suffer from the daily progression of symptoms, holding a different assessment of treatment effectiveness than that considered by the medical professionals. This study summarises the annual reports of the society and the results of periodic surveys conducted by their own. The 20-year annual reports are based on J-MPS membership records since 2005. The three questionnaire surveys were planned, designed, and conducted by the patient association own. Survey I was conducted mainly about treatment and activities of daily living with MPS I, II, IVA, and VI for which ERTs are available. Survey II asked about current symptoms and medical care with MPS IIIA and IIIB. Survey III was focused on ocular complications and skeletal symptoms with all types of MPS.

**Results:**

MPS II is the most common type of membership, and the average age of members is gradually increasing over the year. In MPS, age at diagnosis has decreased compared to previous surveys. Even though enzyme replacement therapy and hematopoietic stem cell transplantation have improved symptoms, there is a slight discrepancy between improvement and patient satisfaction. The degree of need for assistance in life varies with the disease type. Decreased bone mineral density and retinal pigmentary degeneration may be underestimated.

**Conclusions:**

This research is not physician-led evaluations, but patient-driven surveys, which is significant in that it focuses on the problems faced by patients and their families in the current era when enzyme replacement therapies are available. Coordination among patients, families, health care providers, and physicians is important to improve patients’ quality of life.

## Background

Mucopolysaccharidosis (MPS) is a group of lysosomal storage diseases (LSDs) with systemic accumulation of glycosaminoglycans (GAGs) due to defective lysosomal hydrolases that degrade GAGs [1]. MPS is classified into 13 major subtypes (I, II, IIIA, IIIB, IIIC, IIID, IVA, IVB, VI, VII, IX, X and Plus Syndrome) by molecular defect and further subtyped by clinical severity. GAGs progressively accumulate in organs such as blood vessels, heart, liver, spleen, cartilage, and central nervous system, and the severity of each type varies according to the genetic variants or degree of residual enzyme activities [2, 3].

In Japan, the association of families of MPS patients (Japan MPS Patient and Family Group, hereafter referred to as J-MPS) was founded in 1986 to promote interaction among MPS patients and their families. This is the largest patient group for all types of MPS in Japan, and now includes patients and families with “orphan” rare LSDs other than MPS for whom patients’ associations do not exist. Since 1997, J-MPS has co-hosted an annual joint symposium with the physicians’ MPS Study Group.

The J-MPS conducts its periodic surveys using the questionnaire method. This study reports the results of surveys. Although many physician-led outcome surveys based on clinical data have been conducted, this report presents the results of a patient-reported outcome study conducted independently by patients. This research is significant in that it focuses on the problems faced by patients and their families in the current era when enzyme replacement therapies are available.

## Methods

### Study design

J-MPS annually updates the number of affiliated members. The J-MPS also periodically conducts fact-finding surveys and questionnaires focusing on specific disease types or clinical topics. Surveys are conducted either by sending questionnaires by postal mail or by using a web-based system. The surveys are designed and conducted primarily by the J-MPS and are open to their members. The purpose and sample composition differed between surveys due to differences in their respective objectives. The consulting physicians assessed and analysed the survey results retrospectively.

### Annual reports of membership of J-MPS (2005–2024)

It is an aggregate of the patients comprising the J-MPS. It uses information on disease type and age extracted from the registry. Patient and families register their basic data upon becoming members of J-MPS and update it as necessary.

### Survey I: MPS I, II, IVA, VI (2017–2018)

Survey I was conducted in two parts, one in April 2017 and the other in September 2018 on J-MPS members with MPS I, II, IVA, and VI for which ERTs are available [1]. The survey was mainly concerned with current treatment and activities of daily living (ADL). ADLs are subjective assessments of daily living set by the J-MPS. The ADL scale used in this study is divided into four categories: Able to live independently without any disability / Able to live independently with some disability / Partial assistance / Full assistance. Surveys were performed via postal mail.

### Survey II: MPS IIIA, IIIB (2022)

Survey II was conducted in February 2022 on J-MPS members with MPS IIIA and IIIB. This was mainly concerned with current symptoms and medical care. Survey was performed via postal mail.

### Survey III: MPS all types (2022)

Survey III was conducted in October 2022 on J-MPS members with all types of MPS. The survey specifically focused on ophthalmological diagnoses and skeletal symptoms. Skeletal symptoms were collected via questionnaires based on the patients’ self-reported symptoms and caregivers’ assessments. Regarding ophthalmological diagnoses, the survey collected the diagnostic results communicated to patients by specialists through patient questionnaires; these were not collected directly from ophthalmologists. Bone mineral density (BMD) was measured at hospitals, and copies of the measurement results held by patients were collected from them with their consent. In addition, a survey was conducted on the social support systems they are currently receiving. The survey was performed via a web tool (Google form).

## Results

### Annual reports of membership of J-MPS

**Fig. 1:**
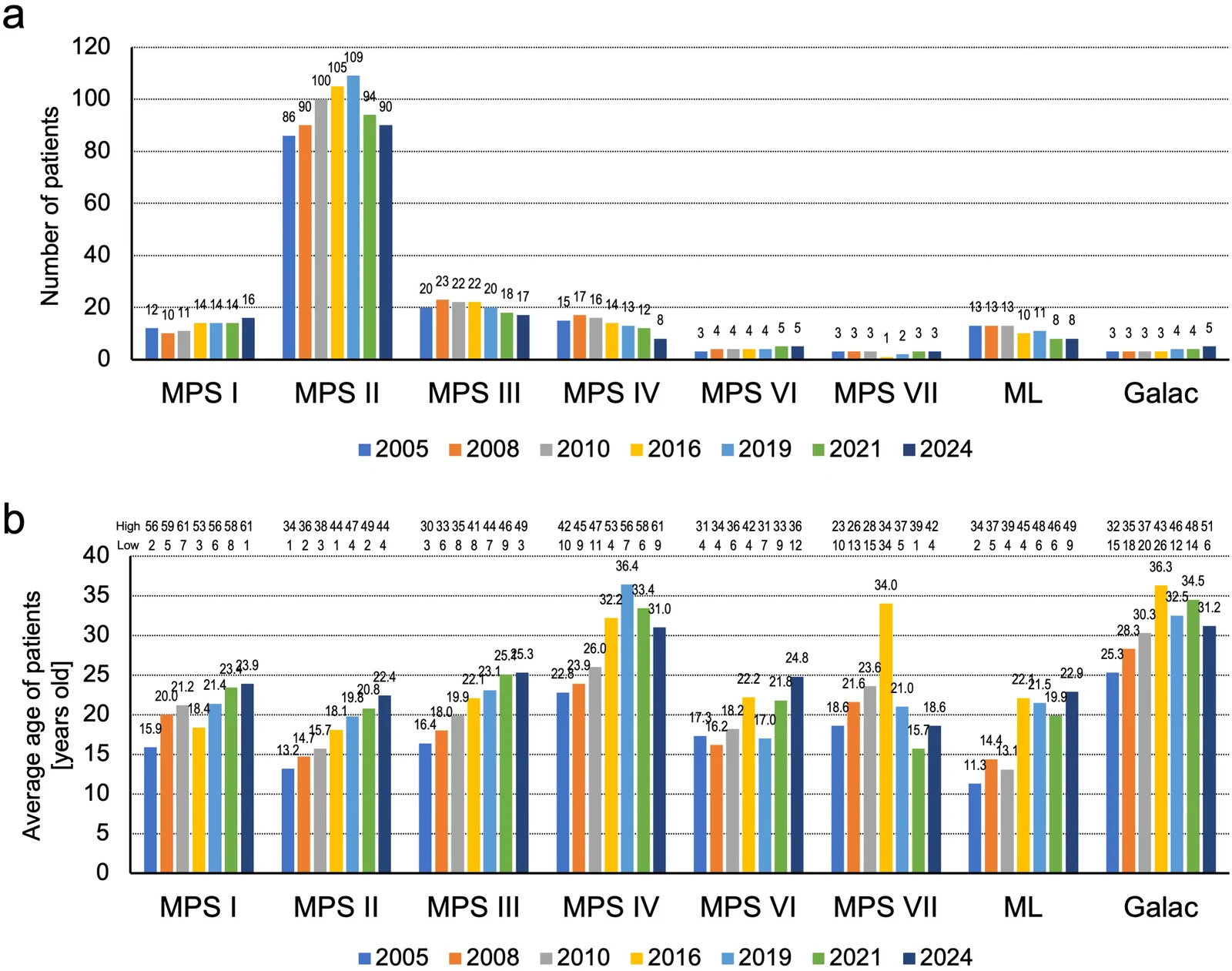
Annual reports of membership of J-MPS (2005–2024). (**a**) Number and (**b**) age of patients in each disease group by year are shown. The highest (High) and lowest (Low) ages in each group are shown above the graph (**b**)

Numbers and ages of the patients in J-MPS are shown in Fig. 1. The latest number of patients as of April 2025 was 156 (MPS I 16; II 82; IIIA 6; IIIB 8; IIIC 2; IV 7; VI 5; VII 3; GM1 gangliosidosis 3; GM2 gangliosidosis 11, sialidosis 1; galactosialidosis 4; mucolipidosis II/III 8). There are no members recorded with MPS IIID, IVB, IX, X and Plus Syndrome. As mentioned before, the J-MPS includes LSD patients for whom no patient associations exist. Figure 1 includes data from MPS I, II, III, IV, VI, VII, mucolipidosis types II/III (ML), and galactosialidosis (Galac) in 2005, 2008, 2010, 2016, 2019, 2021, and 2024. Other LSDs are excluded from this graph because they joined J-MPS after 2016. Figure 1a shows the numbers of patients in J-MPS. The most common type of patient is MPS II in this association, followed by MPS I, III, IV, and ML. Patients of MPS VI, VII, and Galac are rare. The number of participating patients is generally stable but slightly decreasing. The average, maximum, and minimum ages of patients in each disease are shown in Fig. 1b. In MPS I, II, III, IV, and ML, those memberships consist of more than 10 patients, average ages of members showed a tendency of gradual increase.

### Survey I

#### Age at diagnosis in MPS I, II, IVA, and VI patients

**Fig. 2:**
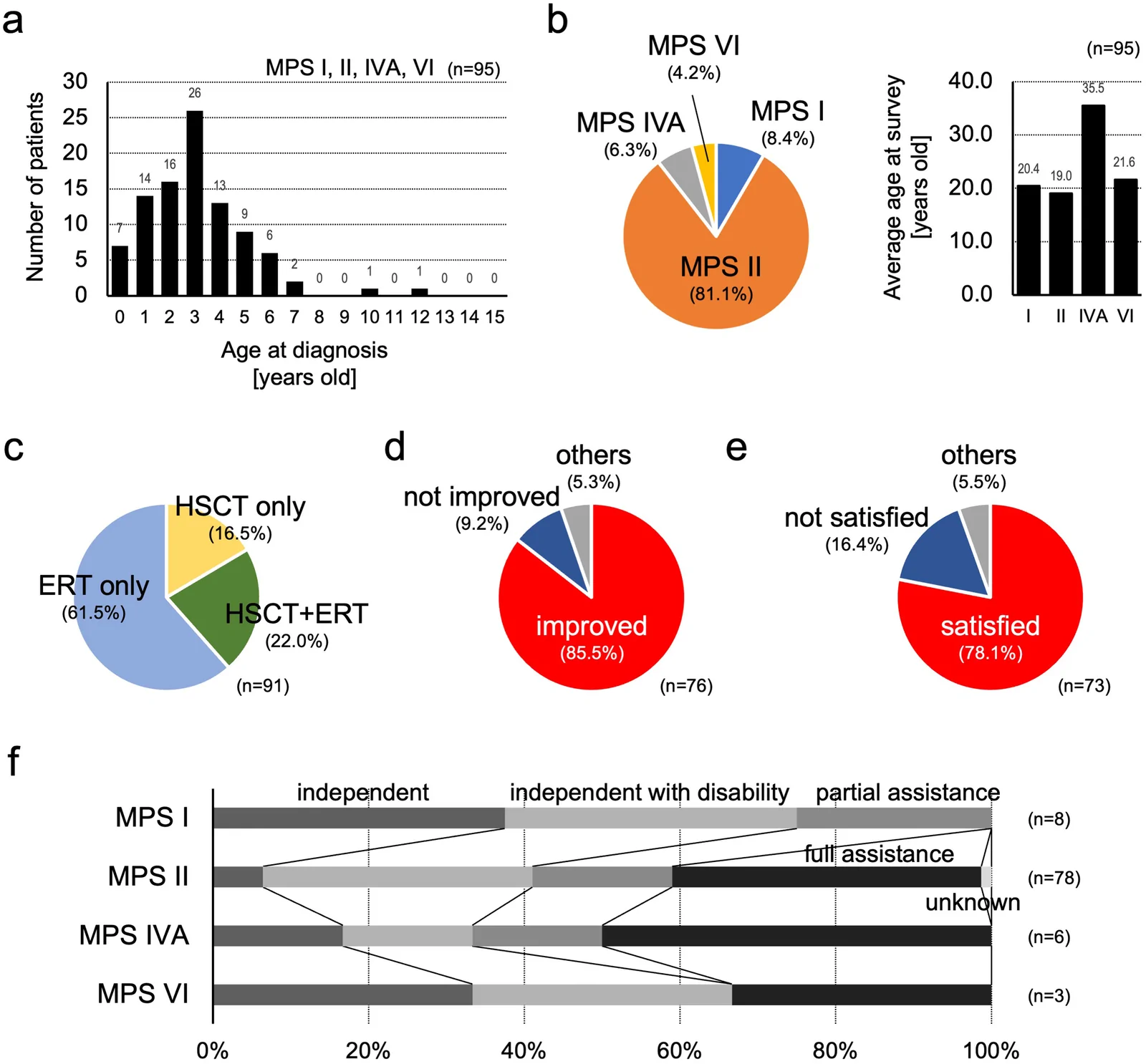
Survey I: MPS I, II, IVA, VI (2017–2018). (**a**) Age at diagnosis. (**b**) Composition of MPS and age at the time of the survey. (**c**) The ratio of patients who have undergone hematopoietic stem cell transplantation (HSCT) and/or enzyme replacement therapy (ERT). Subjective ratings by patients and caregivers regarding (**d**) treatment efficacy and (**e**) satisfaction with these therapies. Others include incomplete answers. (**f**) Activities of daily living

Survey I was a two-part investigation conducted in April 2017 and September 2018 on MPS I, II, IVA, and VI patients. In Japan, enzyme replacement therapy (ERT) for MPS I, MPS II, and MPS VI were approved in 2006, 2007, and 2008, respectively. ERT for MPS IVA started in 2015. Survey I was performed on patients with MPS I, II, VI for which ERTs have been approved for ~ 10 years in Japan—and MPS IVA was additionally included. 142 patients belonging to the J-MPS were surveyed, and responses were received from 95 patients (MPS I 8; II 77; IVA 6; VI 4), resulting in a response rate of 66.9% (95/142). The age at which the disease was diagnosed is shown in Fig. 2a. The results are a sum of all MPS types. The largest number of patients were diagnosed at age 3, followed by age 2 and age 1. 66.3% of all patients were diagnosed by age 3, and 95.8% by age 6; cases diagnosed after age 7 were rare.

#### Improvement and satisfaction with treatment in MPS I, II, IVA, and VI patients

The analysis was limited to MPS I, II, IVA, and VI, where ERT is available. Figure 2b shows the composition of each group and age at the time of the questionnaire survey. It can be seen that MPS II accounts for the majority of cases in Japan. The survey asked about treatment related to special therapies, such as hematopoietic stem cell transplantation (HSCT) and/or ERT, other than symptomatic treatment. 61.5% of patients received only enzyme replacement therapy, 16.5% received only HSCT, and 22.0% received both (Fig. 2c).

Subjective ratings by patients and caregivers regarding treatment efficacy and satisfaction with these therapies were also investigated. 85.5% of patients reported some improvement in their symptoms as a result of these treatments (Fig. 2d). On the other hand, 78.1% of patients were satisfied, and 16.4% were not satisfied with their current treatment (Fig. 2e).

#### ADL of MPS I, II, IVA, and VI patients

The results of the survey on patients’ degree of living autonomy (Fig. 2f) showed that MPS I patients could lead their lives with partial assistance, while some MPS II, IVA, and VI patients required full assistance. Especially in Japan, few MPS II patients live fully independently, and the results of this study indicate that many of them have the severe type.

### Survey II

#### Self-reported symptoms in patients with MPS III

**Fig. 3:**
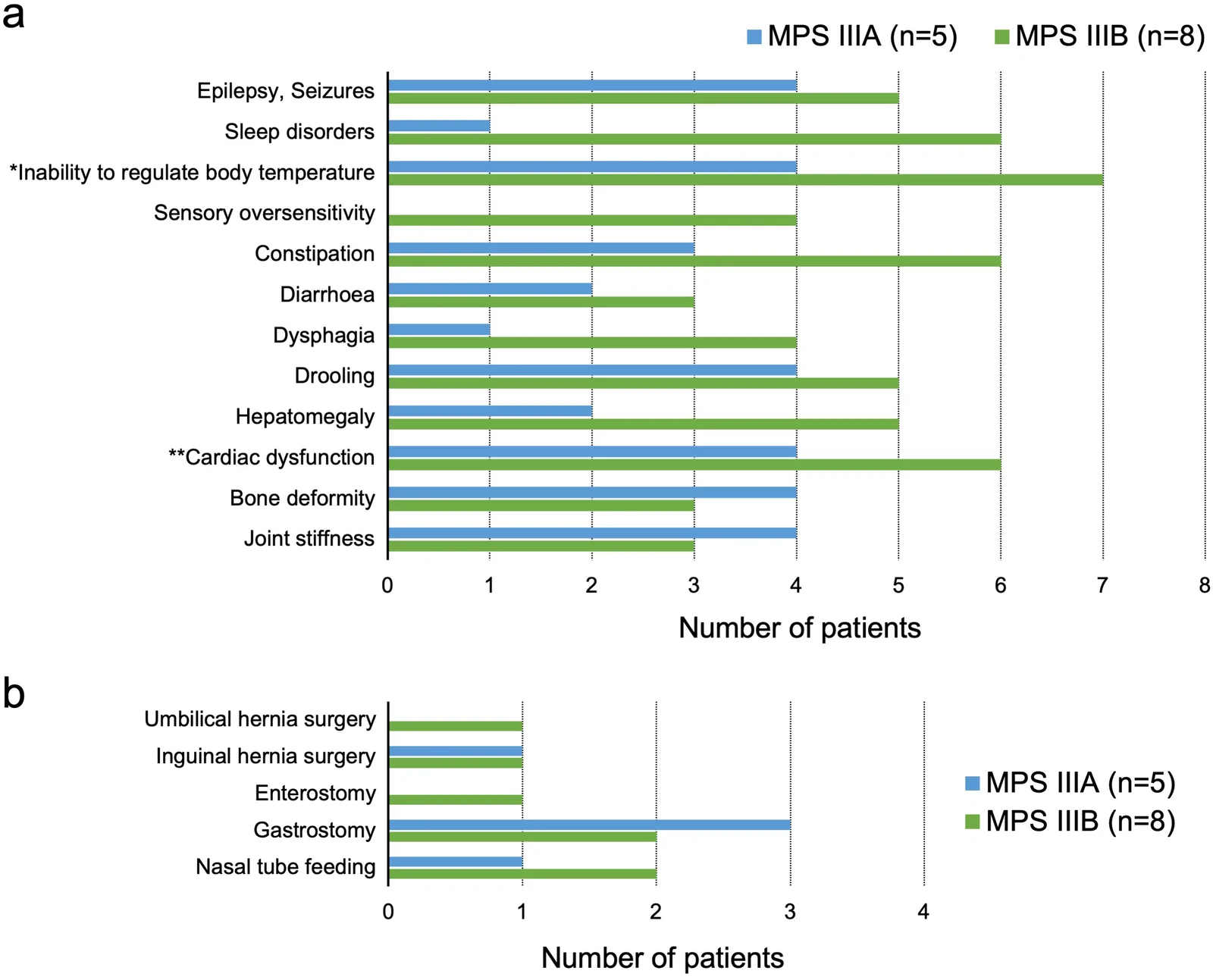
Survey II: MPS IIIA, IIIB (2022). (**a**) Physical symptoms and (**b**) therapeutic interventions other than symptomatic treatment are shown. *Hyperthermia; **Valvular disease, Myocardial hypertrophy, etc

A survey on MPS type IIIA and type IIIB was conducted in 2022. Survey II was conducted in connection with a pharmaceutical company’s development of a new ERT for MPS III, with the aim of exploring patient-reported outcomes that would be useful as clinical trial endpoints. A small number of patients participated in the study, 13 in total 14 patients were surveyed, with a response rate of 92.9% (13/14, IIIA *n* = 5 and IIIB *n* = 8). Physical symptoms are shown in Fig. 3a. Seizures, sleep disorders, hepatomegaly, and skeletal symptoms, which are reported to be common in MPS III patients, were present. Patients also suffer from symptoms related to the nervous system, such as impaired temperature regulation and hypersensitivity, as well as gastrointestinal symptoms such as constipation and diarrhoea. Other therapeutic interventions besides symptomatic treatment, such as hernia surgery, enterostomy, gastrostomy, and tube feeding are shown in Fig. 3b. Many patients need support regarding nutritional intake.

### Survey III

#### Population characteristics of the survey III

Survey III was conducted on all types of MPS patients of J-MPS in 2022. 147 patients belonged to the J-MPS and 77 responses were obtained with a response rate of 52.4% (77/147). Survey III was performed based on the results of previous Surveys I and II. In particular, based on open-ended comments from Surveys I and II, attention was drawn to ocular complications and osteoporosis, and copies of clinical examination data were collected and evaluated. Table 1 shows the characteristics of the population of 77 patients in this survey. In this survey, the severity of the disease was also investigated in each MPS and tabulated separately. The most common type of MPS in this survey was MPS II, accounting for two-thirds of the total. The high prevalence of the severe form among MPS II types is also consistent with previous surveys. The average age of the patients with the severe form of MPS II was lower than that of the attenuated form.

**Table 1:** Survey III: MPS all types (2022). Population characteristics

Disease	Type	Number total (F/M)	Age [years] mean ± sd (median)	Height [cm] mean ± sd	Weight [kg] mean ± sd
MPS I	H (severe)	3 (3/0)	14.0 ± 3.7 (13)	126.0 ± 5.9	29.7 ± 5.2
	S (attenuated)	3 (1/2)	42.3 ± 13.5 (42)	143.0 ± 14.0	47.7 ± 10.0
MPS II	severe	37 (1/36)	17.6 ± 6.7 (16)	138.1 ± 13.1	40.5 ± 10.3
	attenuated	12 (0/12)	26.4 ± 8.5 (22)	148.9 ± 15.1	50.6 ± 8.8
	unknown	3 (0/3)	10.3 ± 8.3 (6)	116.7 ± 23.2	34.5 ± 23.0
MPS III	A	3 (0/3)	41.3 ± 6.6 (45)	155.7 ± 7.7	38.3 ± 5.2
	B	4 (0/4)	19.3 ± 8.3 (18)	137.3 ± 10.2	29.9 ± 6.6
	C	2 (2/0)	34.5 ± 7.5 (34.5)	140.0 ± 6.0	40.1 ± 10.1
MPS IVA	severe	2 (1/1)	52.0 ± 7.0 (52)	117.5 ± 17.5	33.5 ± 14.5
	unknown	1 (0/1)	6.0	94.0	15.6
MPS VI	severe	4 (1/3)	19.5 ± 8.6 (17.5)	123.4 ± 13.6	32.6 ± 11.7
	attenuated	1 (1/0)	34.0	135.0	39.0
MPS VII	severe	1 (0/1)	2.0	75.0	10.5
	unknown	1 (0/1)	7.0	111.0	18.0

F, female; M, male; sd, standard deviation

#### Ophthalmological diagnoses and skeletal symptoms

Table 2 shows the results of the survey of ERT and HSCT, ophthalmological diagnoses, skeletal symptoms, and social supports received for each MPS type. Since this survey was based on spontaneous descriptions, it is not possible to distinguish between cases with no symptoms and those with symptoms not described. Skeletal symptoms can be assessed by the patient or caregiver, but ocular symptoms require a professional diagnosis by an ophthalmologist. Many patients had not visited an ophthalmologist. Short stature was observed in all forms of the disease.

**Table 2:** Survey III: MPS all types (2022). Ocular symptoms, skeletal symptoms, and receiving social supports

		I H sev	I S atten	II sev	II atten	II unk	III A	III B	III C	IVA sev	IVA unk	VI sev	VI atten	VII sev	VII unk
**Number of patients**		3	3	37	12	3	3	4	2	2	1	4	1	1	1
**Therapy**															
	ERT	1	2	19	9	3	0	0	0	1	1	3	1	1	0
	HSCT	0	0	6	1	0	0	1	0	0	0	0	0	0	0
	ERT+HSCT	2	0	12	2	0	0	0	0	0	0	0	0	0	0
**Ophthalmological diagnoses**															
	Decreased visual acuity	3	2	3	2	0	0	0	0	0	0	1	1	0	0
	Cataract	0	0	2	0	0	0	0	0	0	0	0	0	0	0
	Corneal clouding	3	3	1	0	0	0	1	0	2	0	4	1	0	0
	Glaucoma, increased intraocular pressure	0	2	3	0	0	1	1	0	1	0	1	1	0	0
	Nyctalopia	0	3	0	0	0	0	0	1	0	0	1	0	0	0
	Retinal pigmentary degeneration	0	1	2	3	1	0	0	0	0	0	1	0	0	0
**Skeletal symptoms**															
	Joint pain	0	0	5	3	1	0	0	0	1	0	0	1	0	0
	Scoliosis	2	2	14	4	0	2	1	1	1	1	1	0	1	0
	Hand numbness	0	2	2	3	1	0	0	0	1	0	0	1	0	0
	Short stature (average Z-score)	-3.5	-4.0	-3.6	-3.7	-1.9	-1.8	-4.1	-3.3	-8.3	-5.8	-5.9	-4.2	-2.7	-3.3
**Receiving social supports**															
	Medical rehabilitation handbook*	1	0	37	1	2	2	4	2	0	0	0	0	1	1
	Physical disability certificate**	1	3	27	8	2	3	3	1	2	1	2	0	1	0
	Mental disability certificate***	0	0	0	0	0	0	0	0	0	0	0	0	0	0

In Japanese: * Ryoiku Techo, **Shintai Shogaisha Techo, ***Seishin Shogaisha Fukushi Techo. sev, severe type; atten, attenuated type; unk, unknown type

#### Receiving social supports

The survey III also asked whether patients are receiving Japan’s social welfare support systems for persons with disability [4]. Many patients received the ‘medical rehabilitation handbook (Ryoiku Techo)’ and the ‘physical disability certificate (Shintai Shogaisha Techo)’, but none received the ‘mental disability certificate (Seishin Shogaisha Fukushi Techo)’ (Table 2).

#### Bone mineral densities (BMD)

The data for the 11 patients who agreed to provide BMD test results are listed as a case series (Table 3). Ten of the 11 patients for whom BMD data were available showed reduced BMD compared to normal values. With the exception of MPS III, in which severity was not assessed, all of the cases with particularly low BMD Z-scores, less than − 2.0 (Cases 3, 4, and 10), were patients with severe forms of the diseases.

**Table 3:** Survey III: MPS all types (2022). Bone mineral density

						Bone mineral density (BMD)			
	Disease type	Therapy	Age [years]	Sex	Height [Z-score]	Measure point	BMD [g/cm2]	BMD [of *N*]*	BMD [Z-score]
1	MPS I H (severe)	ERT+HSCT	13	F	-3.5	L2-4	0.866	0.87	-1.0
2	MPS I S (attenuated)	−	59	M	-1.8	L2-4	0.891	0.82	-1.5**
3	MPS II (severe)	ERT	9	M	-3.6	L2-4	0.485	0.74	-3.2
4	MPS II (severe)	ERT+HSCT	21	M	-10.1	L1-4	0.511	0.53	-3.5
5	MPS II (attenuated)	ERT	20	M	-0.1	antebrachial	0.448	0.88	NA
6	MPS II (attenuated)	ERT	36	M	-5.7	L2-4	1.098	0.96	-0.2
7	MPS IIIA	−	47	M	-1.5	L2-4	1.176	1.16	1.2
8	MPS IIIC	−	42	F	-2.2	L1-4	0.604	0.61	-3.2
9	MPS VI (severe)	ERT	15	F	-5.3	L2-4	0.659	0.67	NA
10	MPS VI (severe)	ERT	20	M	-4.5	L2-4	0.653	0.62	-4.1
11	MPS VI (attenuated)	ERT	34	F	-4.2	L2-4	1.061	0.89	-0.9

Z-score; transformed value so that the mean is 0 and the standard deviation is 1 in age and sex-matched normal controls. *of N, the relative value of age and sex-matched normal controls; **T-score; −, Neither HSCT nor ERT; NA, not available

## Discussion

J-MPS is an association of patients and families with the largest number of MPS patients in Japan. The composition of the membership is shown in Fig. 1a; in 2024, 16 (10.2%) Type I patients, 90 (59.2%) Type II patients, 17 (11.2%) Type III patients, 8 (5.3%) Type IV patients, 5 (3.3%) Type VI patients, and 3 (2.0%) Type VII patients. This is not significantly different from the report by Orii et al. [5] for the period 1969–2000. Regarding the age of diagnosis, in the previous report [5], 11.3% of cases were diagnosed by the age of 2 years, and 48.2% by the age of 6 years, whereas 38.9% and 95.8%, respectively, in the present report, although it is limited to MPS I, II, IVA and VI (Fig. 2a). It can be evaluated that the age of diagnosis has become much younger, which may be related to the increased diagnostic momentum following the approval of ERT for several MPS. Increased awareness and the establishment of patient registries, combined with the requirements for access to treatment, are also thought to have contributed to this trend. The severe form of MPS II is the most common in Japan and East Asian countries [6]. In Japan, hematopoietic stem cell transplantation has been relatively commonly done for this disease (Fig. 2c). HSCT has been performed on MPS patients [7], but there were ethical concerns in HSCT on MPS II due to limited benefits following transplantation [8]. Tanaka et al. reported the efficacy of HSCT for MPS II, and concluded that HSCT is worthwhile for MPS II patients in the early stages [9].

Physician- or pharmaceutical company-initiated surveys sometimes focus on parameters to obtain regulatory approval, such as adverse effects of drugs and objective parameters like range of motion of joints, hepatosplenomegaly, 6-minute walk, and decreased GAG, as well as on survival [10]. 85.5% of patients reported some improvement in symptoms as a result of ERT or HSCT (Fig. 2d); however, slightly fewer patients—78.1%—were satisfied with their treatment, while 16.4% expressed dissatisfaction (Fig. 2e). A survey conducted in German-speaking cohort [11] also reported a discrepancy between the received treatment and the level of likelihood of favoring that treatment, suggesting that the current treatments, ERT and HSCT, are not quite perfect. The potential persistence of symptoms that are difficult for patients may be related to independence in life, skeletal symptoms, and ocular symptoms [12, 13]. A decrease in BMD has been reported in several MPS types [14]. Of the 11 patients for whom BMD data were provided, 10 showed reduced bone mineral content compared to normal values (Table 3). The severity of osteoarticular symptoms and short stature does not necessarily correlate with decreased bone mineral content. Therefore, it is important to actively evaluate BMD even in cases in which osteoarticular symptoms are not visually noticeable, in order to prevent unintentional bone fractures. On the other hand, assessment of ocular symptoms is complicated. If the patient is unable to communicate effectively, it is extremely difficult for their family to interpret. The presence of corneal clouding or cataracts makes fundus examination difficult. In addition, it is also difficult to perform a calm examination in cases with strong central nervous system symptoms that do not receive instructions, and as a result, accurate evaluation is impossible in small medical clinics (Table 2). In such cases, consulting a large specialist metabolic medical centre several times a year to undergo a comprehensive evaluation, including an eye examination, should be recommended. In MPS, retinal degeneration is thought to be caused by significant GAG deposition in the outer retinal layers (photoreceptor cells) [15]. Retinal pigmentary degeneration may be underestimated in MPS. Because of the slow progression of symptoms and the difficulty of detection, it is important to actively suspect and evaluate the presence of the disease [16, 17]. Although there may be no fundamental treatment for retinal pigmentary degeneration, some patients use eye drops that have anti-inflammatory or neuroprotective effects. Most importantly, clarifying the presence of symptoms is significant in that it raises those symptoms as targets for future development of treatment.

In Japan, when receiving public assistance due to intellectual or physical disabilities, it is necessary to have a certificate issued [4]. The ‘medical rehabilitation handbook’ is issued in principle to persons under the age of 18 who have been assessed as having an intellectual disability. The ‘physical disability certificate’ is issued to those with permanent physical disabilities. Therefore, obtaining such certifications is an indication of the need for public assistance due to these disabilities. As shown in Table 2, many patients with severe forms of MPS I and II have both a medical rehabilitation handbook and a physical disability certificate, while many patients with attenuated forms have only a physical disability certificate. This suggests that the severe type includes intellectual disability, while the attenuated type is exclusively physical. In MPS III, there are many cases of obtaining both a medical rehabilitation handbook and a physical disability certificate, probably because of the severe intellectual disability. Most MPS IV patients have only a physical disability certificate and do not have a medical rehabilitation handbook, however, half of them need full assistance (Fig. 2f). Thus, it can be seen that the need for assistance increases not only due to intellectual disabilities but also due to physical disabilities. The ‘mental disability certificate’ is issued to individuals who face certain limitations in their daily and social lives due to a mental disability (excluding intellectual disabilities), with the aim of promoting their social reintegration and participation. The reason why none received it is thought to be that, as MPS patients have already obtained a ‘medical rehabilitation handbook’ or a ‘physical disability certificate’ due to intellectual or physical disabilities, there is no benefit in obtaining a ‘mental disability certificate’ in addition to the aforementioned handbooks, even if they develop a mental disability. The handbook (Techo) system in Japan is unique and cannot be generalized in the world. Social support for patients requiring medical care in Japan is, in some respects, more generous than in other countries. With advances in treatments, it is valuable to raise discussions about the symptoms experienced by patients in the chronic phase and the appropriate form of support for them. For patients in the chronic phase, the status of obtaining a Techo may serve as an indicator that clearly reflects their current physical condition. The status of obtaining social support is considered worthy of attention.

This survey is a compilation of information described by the patients themselves and their caring family members. It is thought that subjective symptoms are not appropriately assessed in patients who are unable to communicate. In addition, some of the information may be subjective or contain erroneous information. Generally, in the field of medicine, severe MPS II is defined as a form of the disease accompanied by central nervous system symptoms. The criteria of severity differ between medical doctors and patients. This discrepancy was realized by the present survey, which was patient-led rather than doctor-led, and is an important issue for discussion. A previous paper of MPS II from Japan [18] reported that 41 among 65 patients (63.1%) showed a severe form. In another recent survey conducted at Japanese medical institutions, among 53 MPS II patients with confirmed disease types, 32 were classified as severe, resulting in a severe type ratio of 60.4% [19]. On the other hand, in Survey III of the present study, based on patients’ self-reports, among 49 MPS II patients with confirmed disease types, 37 (75.5%) answered as severe (Table 3). Of course, there are cases where patients report the type of disease they have been told by their doctors, but there are also cases where they make judgments based on their current physical condition. Since the disease is progressive, patients’ self-assessments may change over time, and the severity may be reported as higher than at the time of diagnosis. It is an interesting point in the present research that needs to be carefully examined.

This study analyses the treatments that the survey responders received. However, detailed information on the duration of HSCT and ERT, as well as the timing of switching or combining these therapies, is not available. Furthermore, there is no data on the engraftment rate (chimerism) of transplanted cells after HSCT. We believe these are the limitations of a patient survey study. On the other hand, it should be noted that complex questionnaire items may reduce the response rate and may distort the population. The especially high response rate in Survey II (92.9%) is thought to be due to the motivation to participate in the development of new treatments. The reason for relative low response rate for Survey III (52.4%) is thought to be the large number of questions and the free-form format. The response rate in Survey I was 67.4%. According to a recent study, the response rate of the survey on COVID-19 in Japanese Fabry disease patients was 55.8% [20], although COVID-19 is a topic of great interest. Therefore, the response rate for the surveys in this study is considered to be not low, which is also considered a patient-driven survey benefit. Survey II showed a very high response rate with its checkbox format, but the selection of these items was based on preliminary survey results within the patient group.

## Conclusions

This study has the unique feature of retrospectively summarizing the results of a questionnaire survey that was planned and conducted entirely by the patient association, from conception to implementation. While there are several limitations in terms of consistency in planning, the target disease, or methodology, because the survey was conducted from the patient’s perspective to identify their current issues, it still holds value in highlighting intriguing points that are not typically addressed from a healthcare provider’s perspective. Such patient-reported outcomes have attracted considerable attention in recent years. It is also important to recognise the differences in social support systems and the current situations of healthcare provision between countries. If patient groups and academia can work hand in hand to conduct research that integrates and analyzes both subjective evaluations, such as this study and objective evaluations, such as enzyme activity and medical expert findings based on medical records, more meaningful results can be produced. For this purpose, voluntary activities by patient associations are very important.

## Data Availability

All relevant data that supports the findings of the study are within the manuscript.

## Abbreviations

ADL: Activities of daily living
BMD: Bone mineral density
ERT: Enzyme replacement therapy
GAGs: Glycosaminoglycans
Galac: Galactosialidosis
HSCT: Hematopoietic stem cell transplantation
LSDs: Lysosomal storage diseases
ML: Mucolipidosis types II/III
MPS: Mucopolysaccharidoses
